# How to Reach a Regional Cooperation Mechanism to Deal With the Epidemic: An Analysis From the Game Theory Perspective

**DOI:** 10.3389/fpubh.2021.738184

**Published:** 2021-10-14

**Authors:** Hualei Yang, Yuanyang Wu, Yidan Yao, Siqing Zhang, Shuo Zhang, Lin Xie, Zhiyun Li, Lili Tang

**Affiliations:** ^1^School of Public Administration, Zhongnan University of Economics and Law, Wuhan, China; ^2^Institution of Population and Labor Economics, University of Chinese Academy of Social Science, Beijing, China; ^3^College of Politics and Public Administration, Qingdao University, Qingdao, China; ^4^College of Chemistry and Chemical Engineering, Yantai University, Yantai, China

**Keywords:** the COVID-19, cooperation, game theory, the epidemic, income distribution

## Abstract

The outbreak and persistence of COVID-19 have posed a great threat to global public health and economic development. The continuous economic deterioration has been intensified due to the continuous prevention and control measures, such as closed management. Insisting on the prevention of the epidemic or economic restart has become a dilemma for all countries. Epidemic prevention is not only the main behavior of a single country but also a common problem faced by all countries in the region. Continuous prevention measures will affect economic development, but an early restart of the economy is faced with the recurrence of the epidemic. To avoid the emergence of prisoner's dilemma in the governance of the epidemic, each country cannot make decisions with its optimization, and so it is necessary to build a regional cooperation mechanism to achieve the overall optimization of the economy and prevent the epidemic. Based on the game theory, we analyzed the behavior of countries when carrying out regional cooperation to govern the epidemic and put forward specific cooperative income distribution schemes according to the different attributes of the countries. Our results showed that in the presence of population mobility, regional cooperation to govern the epidemic can minimize the total number of infected people and maximize the overall utility of the region, which was significantly better than the overall benefits of the region in the case of non-cooperation. However, in detail, the smaller the difference of preference for preventing and controlling the epidemic between the two, the more likely it is to lead to a win-win situation. Otherwise, there will be one with damaged interests. When damaged interests appear, the appropriate distribution of cooperative income to the country with a small economic scale and low preference in preventing the epidemic is more conducive to the achievement of cooperative mechanisms and the realization of a win-win situation in the region.

## Introduction

Since the outbreak of the epidemic, COVID-19 has spread to nearly 200 countries and regions worldwide, and the number of infected persons has reached about 10,000,000, making it a global public health problem. For a period of time, traffic closure, business stop, home isolation, and other prevention and control measures have become a powerful antiepidemic weapon for countries to contain the epidemic (1–3). However, the long-term blockade measures are followed by heavy economic costs, and the health crisis may turn into a financial crisis (4). In Asia, as the main driving force of global economic growth, the value of the stock market of China fell sharply when China restarted its economy. The Shanghai Composite Index fell by 7.7%, and its market value shrank by about US $375 billion, the biggest 1-day decline since August 2015 (5). Similarly, the outbreak of COVID-19 paralyzed all kinds of economic activities and sectors in Indonesia, and the quarterly economic growth dropped by 2.41%, which was a significant decline (6). In Europe, due to the restrictions on labor migration brought about by the social segregation policy, the immigration of migrant workers will be refused. In 2020, the GDP of Italy may drop by 43%, the Netherlands 45%, Spain 37%, and Switzerland 200% (7). In the United States, the spread of the epidemic has brought unprecedented impact on the stock market and economic policies. The stock market volatility in the United States is equal to or higher than that in October 1987, December 2008, and December 1929 (8). There are 1,100 oil companies facing the risk of bankruptcy, the number of unemployed people has reached a new record, and the economic contraction may reach about 6.1%.

Faced with such a heavy economic cost, many regions are trying to adopt the policy of economic restart. Take the United States as an example, in April, the federal government announced that it would restart the economic plan. Some local states could take the lead in opening large-scale venues including restaurants, cinemas, sports venues, and religious places. These relaxation measures promoted the temporary improvement of store sales and economy but also created opportunities for the rebound of the epidemic. In many states where the epidemic was not serious, the situation has taken a sharp turn, and the epidemic data has soared day after day. Among the 29 states where the epidemic rebounded, the number of infected cases in at least 13 states increased by more than 50%, whereas the number of cases in the other 16 states increased by 10–50%. For states that advocate economic restart, economic restart means relaxing epidemic prevention and control measures. The emergence of clustering activities will reduce the effect of prevention and control, leading to an increase in the number of infected people. Population flow between states is bound to reduce the effect of prevention and control in other states and affect the overall effect of prevention and control in the region. For the states that advocate strengthening prevention and control, the prevention and control measures of maintaining social distance and stopping production have seriously affected the normal order of the market economy. So, unemployment, declining output value, and economic downturn have become the main problems they are facing. At the same time, the spread of the number of infected people has an externality. Decision-makers should not only consider their benefits but should make decisions based on the perspective of maximizing the overall regional benefits to avoid falling into the tragedy of the commons. To solve the dilemma between economic restart and epidemic prevention and control, it is necessary to build a regional cooperation mechanism.

In view of the existing studies, many scholars have made active exploration from the perspective of epidemic prevention and control (9–11). Especially in the last several years, in the field of social physics and statistical physics, there were some studies combining epidemiology with evolutionary game theory. In the *Sociophysics Approach to Epidemics*, Tanimoto systematically discussed the application of evolutionary game theory in epidemiology, containing two-player and two-strategy games, multiplayer games, and social dilemmas (12). The evolutionary game theory helped us to analyze human decision-making and social attitudes shared through a virtual network, which was an important part of social physics. For example, the game theory was widely used in the analysis of vaccination decisions. Bauch and Earn (13) investigated the feedback between individual vaccination decisions and population-level processes that determine vaccine uptake and herd immunity for endemic disease and thought that vaccination decisions were strongly influenced by incorrect risk perception. Piraveenan et al. emphasized that decision-making under uncertainty and imperfect information, and with only conditionally optimal outcomes, was a unique forte of established game-theoretic modeling. The game theory and social network models were used to study the vaccination uptake in their study, which became the key factor that would determine our success in containing COVID-19 (14).

The choice of the behavior of people in the epidemic was also important to control the epidemic. Evolutionary game theory and public goods games offered an important framework to understand human choice during pandemics (15). In the absence of empirical data about the COVID-19, policymakers must turn to epidemiological modeling to evaluate the actions for responding to the pandemic. Kabir and Tanimoto combined compartmental epidemiological models with the concept of behavioral dynamics from evolutionary game theory (EGT) and concluded that the effects of shield immunity and economic shutdowns were complementary, and governments should pursue them in tandem (16). Then, the behavior of wearing a mask was further analyzed by Kabir et al. They developed a new intervention game model that combined the mathematical models of epidemiology with EGT, which quantified how people use mask-wearing and related protecting behaviors that directly benefit the wearer and bring some advantage to other people during an epidemic (17). In terms of the effect of social distancing and self-quarantine, Ngonghala et al. developed a model framework that integrated COVID-19 transmission dynamics with a multistrategy evolutionary game approach of individual decision-making and found that social distancing played a major role in reducing the burden of the disease compared to self-quarantine (18).

In addition, the strategies among governments were also explored. Kabir et al. proposed a novel epidemic model associated with behavioral dynamics under the EGT by considering the two-body system, and they agreed that the funds spent on the individual level as an “emergency relief-package” can reduce the infection and improve quarantine policy success (19). Wei et al. established transmission frequency equations, which combined the interaction strategies and the evolutionary game analysis of the actions taken by the government and the public, and found that the emergency response strategy adopted by the government in the early days of the epidemic can effectively control the spread of the epidemic (20). All the above studies combined epidemiological research with game theory, which provided enlightenment for current and future epidemic prevention. Therefore, based on the previous research, this study further studied the behavior of the countries for promoting cooperation in response to the COVID-19 in a region, and the distribution of cooperative income combined the epidemiology with two-body game theory.

## Methods

### Utility Function Setting

During the outbreak of COVID-19, a region first faced the health crisis caused by the spread of the virus. The intervention measures, such as isolation to prevent the spread of the epidemic, will reduce the number of infected persons, and control the spread of the epidemic, that is the prevention and control utility. Second, economic development can bring economic benefits to the region, and that is economic utility. Then suppose that there are two countries in a region, and there is a flow of infected cases between them, which affects the prevention and control effect of the two countries. Considering the setting of function from Wang et al., we set the utility function of each country to include two parts (21). The two parts included economic utility and prevention and control utility:


(1) $$\begin{array}{r} U_{i}=f\left(Y_{i}\right)+P_{i}g\left(H_{i}^{s}\right) \end{array}$$

where *i* = 1, 2 denotes country 1 and country 2, respectively; *Y*_*i*_ denotes economic output, $H_{i}^{s}$ denotes prevention and control effect; *f*(*Y*_*i*_) is economic utility, $g\left(H_{i}^{s}\right)$ is prevention and control utility, and *P*_*i*_ indicates the preference of “*i*” country for the prevention and control of the epidemic.

Set the relationship between economic output and the number of infected people. Before the development of vaccines, experience has shown that isolation, cancellation of aggregation activities, and other closed measures will help to curb the increase in the number of infected people, but closed isolation measures will also lead to the cessation of commercial activities and affect economic growth. The less isolation measures, the more frequent the economic activities and the higher the economic output, but at the same time, the more infected people are. So, the economic output is as follows:


(2) $$\begin{array}{r} Y_{i}=Y_{i}\left(H_{i}\right)=\alpha_{i}\times H_{i} \end{array}$$

Where *H*_*i*_ is the number of infected people; α_*i*_ is the economic output created by *i* infected people in the country.

The relationship between the effect of prevention and control and the number of infected people was set. In a region, the prevention and control effect of a country is affected not only by the number of local infected persons but also by the input of infected persons in other countries. In this study, it is assumed that β_*i*_ part of the infected population in one country will stay in this country, and the rest will be imported into the other country through population flow, β_*i*_ ϵ(0, 1). The number of infected people can represent the epidemic prevention and control effect of a country, but there is a complex relationship between them. In this study, the prevention and control effect of a country is simplified as the threshold of the national medical system for the number of infected people minus the total number of infected people in the country, and it is assumed that it is >0. The prevention and control effect was set as follows:


(3) $$\begin{array}{r} H_{i}^{s}=H_{i}^{c}-\left[\beta_{i}\times H_{i}+\left(1-\beta_{j}\right)H_{j}\right] \end{array}$$

Referring to Wang et al. (21), we assumed that the utility brought by economic output and prevention and control effect meets the law of diminishing marginal utility, which is further embodied in the form of a logarithmic function. Therefore, the utility function of a national government can be obtained as follows:


(4) $$\begin{array}{r} U_{i}=\ln\;\left(\alpha_{i}\times H_{i}\right)+P_{i}\times \ln\;\left\{H_{i}^{c}-\left[\beta_{i}\times H_{i}+\left(1-\beta_{j}\right)H_{j}\right]\right\} \end{array}$$

where *j* is the other country, $H_{i}^{c}$ is the threshold of the number of infected people that the *i* country can bear, and $H_{i}^{c}>0$.

## Results

### Decision Making of Two Countries in a Non-cooperative Situation

Considering the situation that the two countries do not cooperate in the epidemic, the two countries independently choose their optimal number of infected people. The utility of the two is as follows:


(5) $$\left\{ \begin{array}{l} U_{1}=\ln(\alpha_{1}\times H_{1})+P_{1}\times \ln\left\{H_{1}^{c}-\left[\beta_{1}\times H_{1}+\left(1-\beta_{2}\right)H_{2}\right]\right\}\\ U_{2}=\ln(\alpha_{2}\times H_{2})+P_{2}\times \ln\left\{H_{2}^{c}-\left[\beta_{2}\times H_{2}+\left(1-\beta_{1}\right)H_{1}\right]\right\} \end{array}\right.$$

Each rational country will choose the optimal prevention and control effect to maximize its utility when given the number of infected people in the other country. The first order conditions (FOC) of the two are as follows:


(6) $$\left\{ \begin{array}{l} \frac{1}{H_{1}}+P_{1}\times \frac{-\beta_{1}}{H_{1}^{c}-\left[\beta_{1}H_{1}+\left(1-\beta_{2}\right)H_{2}\right]}=0\\ \frac{1}{H_{2}}+P_{2}\times \frac{-\beta_{2}}{H_{2}^{c}-\left[\beta_{2}H_{2}+\left(1-\beta_{1}\right)H_{1}\right]}=0 \end{array}\right.$$

From the above formula, we can get the Nash equilibrium number of infected cases in each country in the case of non-cooperation.


(7) $$\left\{ \begin{array}{l} H_{1}^{*}=\frac{\beta_{2}(1+P_{2})H_{1}^{c}-(1-\beta_{2})H_{2}^{c}}{\beta_{1}\beta_{2}(1+P_{1})(1+P_{2})-(1-\beta_{1})(1-\beta_{2})}\\ H_{2}^{*}=\frac{\beta_{1}(1+P_{1})H_{2}^{c}-(1-\beta_{1})H_{1}^{c}}{\beta_{1}\beta_{2}(1+P_{1})(1+P_{2})-(1-\beta_{1})(1-\beta_{2})} \end{array}\right.$$

Especially, when the other parameters of the two countries are the same, the optimal number of infected people in the two countries is the same.


(8) $$\begin{array}{r} H_{1}^{*}=H_{2}^{*}=\frac{H^{c}}{\beta P+1} \end{array}$$

In this case, the utility of the two countries is as follows:


(9) $$\begin{array}{r} U_{1}^{*}=U_{2}^{*}=\left[\ln\;\left(\alpha_{i}\times H_{1}^{*}\right)+P_{i}\ln\;\left(H^{c}-H_{1}^{*}\right)\right]\\ =\left[\ln\;\left(\alpha_{i}\times \frac{H^{c}}{\beta P+1}\right)+P_{i}\ln\;\left(\frac{\beta PH^{c}}{\beta P+1}\right)\right] \end{array}$$

It can be seen from the above formula that the optimal number of infected people in the two countries are affected by the preference coefficient of prevention and control *P*_*i*_, the proportion of infected people who stayed in their own country β_*i*_, and the threshold of the number of infected people $H_{i}^{c}$. First, when other conditions remain unchanged and when the prevention and control preference coefficient of country 1 is larger, the number of infected people will decrease because when the prevention and control preference increase, the prevention and control effect of reducing the number of infected people is greater than the economic effect. So, country 1 will implement more stringent prevention and control measures to reduce the number of infected people. But at the same time, the number of infected people in country 2 in the same region will increase because the decrease of the number of infected people in country 1 will lead to the decrease of the number of infected cases imported from abroad in country 2 so that there is more room for the number of infected people in country 2 to be treated. So, the control measures in country 2 will be relaxed and economic activities will be opened to increase economic output, to maximize its utility.

Second, when other conditions remain unchanged, the larger the proportion of infected people stayed in their own country, the greater the impact of the increase in the number of infected people on itself, and the smaller the impact on the other country, and so the number of infected people in their own country will increase and the number of infected people in the other country will decrease.

Finally, when other conditions remain unchanged, the increase of the threshold of the number of infected people in country 1 means that country 1 has the ability to bear more infected people and has the conditions to relax the prevention and control measures to obtain greater economic benefits. However, due to the import of more overseas infection cases, country 2 will take strict prevention and control measures to reduce the number of domestic infected cases.

### Decision Making of Two Countries in a Cooperative Situation

In the case of cooperation, the behavior of the two countries conforms to the assumption of collective rationality, that is, to choose the prevention and control strategy under the maximum regional overall utility and to realize the number of infected people under the maximum regional utility. The total utility of the region is obtained by summing up the utility of the two countries:


(10) $$\begin{array}{l} U=U_{1}+U_{2}=\ln(\alpha_{1}\times H_{1})+P_{1}\times \ln\{H_{1}^{c}-[\beta_{1}\times H_{1}\\ \;+\left(1-\beta_{2}\right)H_{2}]\}+\ln(\alpha_{2}\times H_{2})+P_{2}\\ \;\times \ln\left\{H_{2}^{c}-\left[\beta_{2}\times H_{2}+\left(1-\beta_{1}\right)H_{1}\right]\right\} \end{array}$$

When the whole region is optimal, the FOC of the above formula is as follows:


(11) $$\left\{ \begin{array}{l} \frac{1}{H_{1}}-\frac{\beta_{1}P_{1}}{H_{1}^{c}-\left[\beta_{1}H_{1}+\left(1-\beta_{2}\right)H_{2}\right]}-\frac{P_{2}(1-\beta_{1})}{H_{2}^{c}-\left[\beta_{2}H_{2}+(1-\beta_{1})H_{1}\right]}=0\\ \frac{1}{H_{2}}-\frac{\beta_{2}P_{2}}{H_{2}^{c}-\left[\beta_{2}H_{2}+\left(1-\beta_{1}\right)H_{1}\right]}-\frac{P_{1}(1-\beta_{2})}{H_{1}^{c}-\left[\beta_{1}H_{1}+(1-\beta_{2})H_{2}\right]}=0 \end{array}\right.$$

We firstly supposed that other parameters of the two countries are the same. The optimal number of infected people in the case of cooperation between two countries is obtained:


(12) $$\begin{array}{r} H_{1}^{**}=H_{2}^{**}=\frac{H^{c}}{P+1} \end{array}$$

By comparing Equations (8) and (12), we can see that the number of infected people in the case of cooperation is less than that in the case of non-cooperation. This difference is mainly caused by the flow of infected cases between countries. In the non-cooperative situation, countries only consider the impact of domestic infection numbers based on their economy, and prevention and control effect, but ignore the external negative effect of imported infected cases on the region, which leads to a larger optimal number of infections. In the case of cooperation, countries need to consider the maximization of the overall utility of the region and consider the negative effect of external infected case input when making the decision in prevention and control or economy restart.

### Analysis on the Change of Regional Overall Utility From Non-cooperation to Cooperation

By analyzing the utility changes of the two countries in the region from non-cooperation to cooperation, it is helpful to analyze the gains and losses of the two countries in the regional cooperation in response to the epidemic, to provide a theoretical basis for the construction of regional cooperation mechanism. The following analysis is mainly from the two countries with the same attributes and two countries with different attributes.

The two countries have the same attributes, which means that the two countries have the same preference for preventing and controlling the infected cases *P*_*i*_, the proportion of infected people staying in their own country β_*i*_, and the threshold of the number of infected people that each country can bear $H_{i}^{c}$. Based on the same attributes of the two countries, we first calculate the utility change from non-cooperation situation to cooperation situation.

In the case of non-cooperation, the two countries choose the optimal number of infected people $H_{1}^{*}$ and $H_{2}^{*}$, and the total utility of the region is:


(13) $$\begin{array}{r} U^{*}=U_{1}^{*}+U_{2}^{*}=2\left[\ln\;\left(\alpha_{i}\times H_{1}^{*}\right)+P_{i}\ln\;\left(H^{c}-H_{1}^{*}\right)\right]\\ =2\left[\ln\;\left(\alpha_{i}\times \frac{H^{c}}{\beta P+1}\right)+P_{i}\ln\;\left(\frac{\beta PH^{c}}{\beta P+1}\right)\right] \end{array}$$

In the case of cooperation, the two countries choose the optimal number of infected people $H_{1}^{**}$ and $H_{2}^{**}$, and the total utility of the region is:


(14) $$\begin{array}{r} U^{**}=U_{1}^{**}+U_{2}^{**}=2\left[\ln\;\left(\alpha_{i}\times H_{1}^{**}\right)+P_{i}\ln\;\left(H^{c}-H_{1}^{**}\right)\right]\\ =2\left[\ln\;\left(\alpha_{i}\times \frac{H^{c}}{P+1}\right)+P_{i}\ln\;\left(\frac{PH^{c}}{P+1}\right)\right] \end{array}$$

Under the same parameters, the change of regional total utility is as follows:


(15) $$\begin{array}{r} \Delta U=U^{**}-U^{*}=2\ln\;\left\{{\left[\frac{\beta P+1}{\beta \left(P+1\right)}\right]}^{P+1}\beta \right\} \end{array}$$

When β = 1, the number of infected people in each country remains completely in its own country, and there is no flow of infected people, that is ${\left[\frac{\beta P+1}{\beta \left(P+1\right)}\right]}^{P+1}\beta =1$ and Δ*U* = 0. Under this situation, the total regional utility of the two countries is the same, and there is no difference in decision-making whether they choose cooperation or non-cooperation. However, when β <1, ${\left[\frac{\beta P+1}{\beta \left(P+1\right)}\right]}^{P+1}\beta$ is a monotonous decrease function of β, and so when β∈(0, 1), ${\left[\frac{\beta P+1}{\beta \left(P+1\right)}\right]}^{P+1}\beta >1$, Δ*U*>0. Therefore, when there is a flow of infected people between the two countries, the choice of cooperation strategy is more effective than the non-cooperation strategy, and the choice of cooperation will generate more utility than the non-cooperation strategy.

To sum up, when the attributes of two countries in the region are the same and there is a flow of infected people, regional cooperation in response to the epidemic can not only achieve the minimum number of infected people but also the overall regional utility is greater than that of non-cooperation. Driven by the minimum number of infected people and the maximum regional overall utility, both countries have the motivation to choose cooperative strategies to jointly prevent and control the epidemic.

In reality, there are differences in attributes between the two countries. When there are differences in preference of countries for preventing and controlling the epidemic *P*_*i*_, the proportion of infected people who stayed in their own country β_*i*_, and the threshold of the number of infected people that countries can bear, the solution is very complex. The following mainly analyzes the utility change through numerical analysis. Considering that in the actual epidemic prevention and control, once the epidemic occurs, countries will immediately take measures to restrict population mobility, the flow of infected people has little influence on the national decision-making in the prevention of the epidemic. Therefore, we focus on the impact of preference in preventing and controlling infected people on the strategy choice of the two countries.

It is assumed that the other attributes of the two countries are the same except for the preference in prevention and control infected people, that is, the proportion of infected people who stayed in their own country and the threshold of the number of infected people that each country can bear are the same, that is β_1_ = β_2_ = 0.5 and $E_{1}^{c}=E_{2}^{c}=E^{c}$. For the degree of preference in the prevention and control of infected people, it is assumed that the degree of preference of country 2 is higher than that of country 1. If the degree of preference of country 1 is *P*, the preference of country 2 is *P*_2_ = *nP*(*n*>1). The utility change of each country can be obtained by selecting the optimal number of infected people in each case:


(16) $$\left\{ \begin{array}{l} \Delta U_{1}=\ln\left[\frac{1}{n}\times {\left(\frac{nP+n+1}{nP+P+2}\right)}^{P+1}\times {\left(\frac{n+1}{n}\right)}^{P}\right]\\ \Delta U_{2}=\ln\left[{\left(\frac{nP+n+1}{nP+P+2}\right)}^{nP+1}\times {\left(\frac{n+1}{n}\right)}^{nP}\right] \end{array}\right.$$

**Figure 1:**
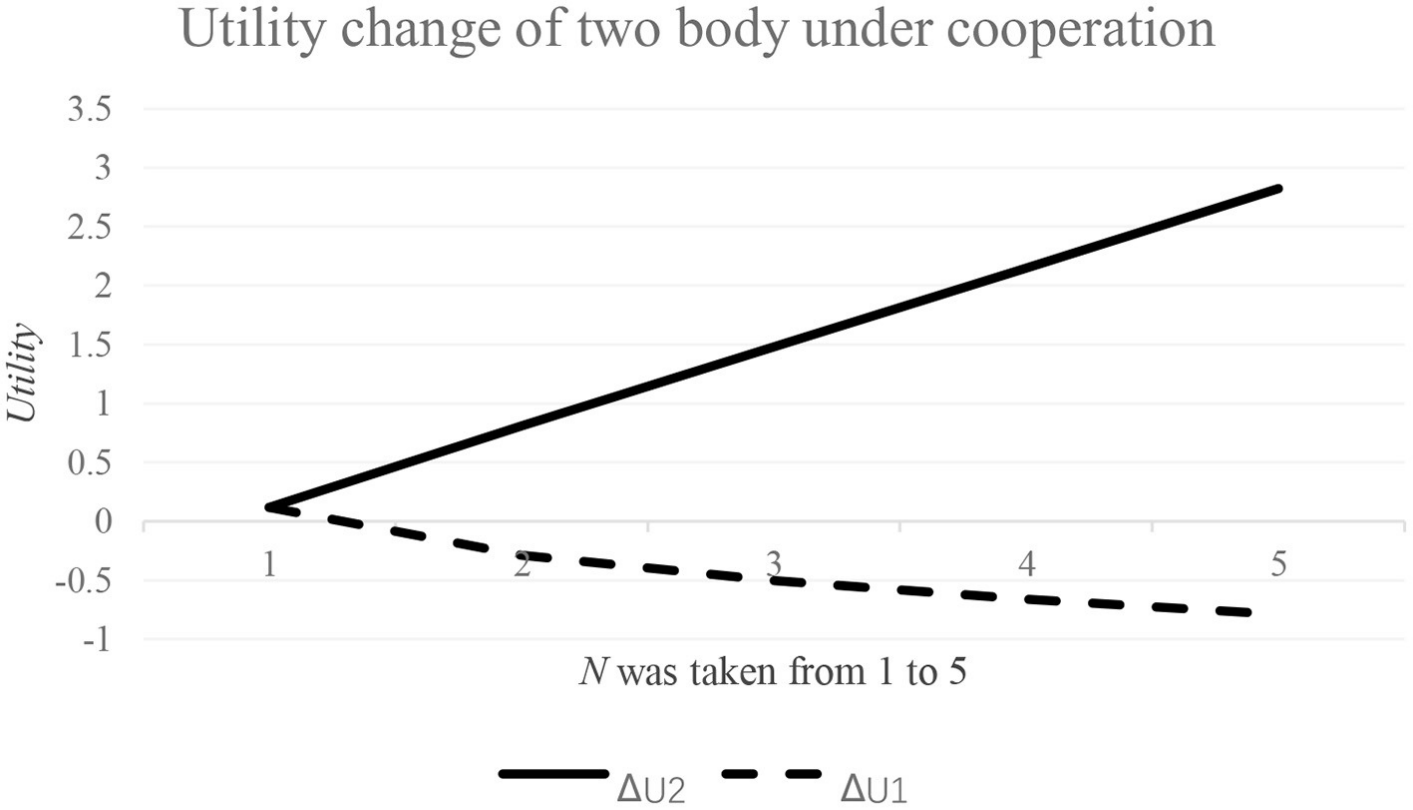
Utility change of two countries in cooperation situation with different *N*.

To analyze the utility change of country 1 and country 2, we need to give specific values of preference coefficient *P* and coefficient *n*. Specifically, *n* values are from 1 to 5 and *P* = 1. The utility changes of the two countries are shown in Figure 1.

As can be seen from Figure 1, in the regional cooperation to cope with the epidemic, with the increase of the difference in preference for prevention and control infected people between the two countries, the income of country 1 in the cooperation gradually decreases, and when *n* = 1.202, the income is 0, and then gradually presents a net loss. On the contrary, with the increase of the difference in preference between the two countries, the income of country 2 increases gradually. The results show that in regional cooperation, when the differences of preference for epidemic prevention and control infected people between the two countries are small, there is a win-win situation, that is, the utility of cooperation is greater than that of non-cooperation. Otherwise, countries with less preference for prevention and control of infected people will be in a disadvantageous position in cooperation, resulting in loss of benefits. This is because, for countries with a large preference for prevention and control, the benefit of controlling infected people increased by the decrease in the number of infected people in cooperation, and this is greater than the economic benefit lost by the decrease in economic output, resulting in an increase in net income. For the countries with less preference for prevention and control of infected people, the benefit of controlling infected cases improved by the decrease in the number of infected people and this is less than the economic benefit lost by the decrease in economic output, so the net income gradually decreases.

Next, we analyzed the impact of the cooperation mechanism on the utility of the two countries under the different proportions of infected patients flowing to the other country. Similarly, we assume that the threshold of infected persons that the two countries can bear is the same, that is $E_{1}^{c}=E_{2}^{c}=E^{c}$. The two countries have the same preference for controlling the epidemic, that is *P*_1_ = *P*_2_, and for the convenience of calculation, we assume *P*_1_ = *P*_2_ = 1. For the proportion of infected patients retained in their own country, we assume that the retained proportion of a country β_1_ = β, the retained proportion in the other country is β_2_ = 1, that is, the other country implemented comprehensive and strict closure measures and did not allow any flow of infected patients. The advantage of this assumption was to realize the asymmetry of retained proportion between the two countries. The utility change of each country can be obtained by selecting the optimal number of infected people in each case:


(17) $$\left\{ \begin{array}{l} \Delta U_{1}=\ln(E_{1}^{**}/E_{1}^{*})+\ln(\frac{E^{C}-\beta E_{1}^{**}}{E^{C}-\beta E_{1}^{*}})\\ \Delta U_{2}=\ln(E_{2}^{**}/E_{2}^{*})+\ln(\frac{E^{C}-E_{2}^{**}-(1-\beta )E_{1}^{**}}{E^{C}-E_{2}^{*}-(1-\beta )E_{1}^{*}}) \end{array}\right.$$

**Figure 2:**
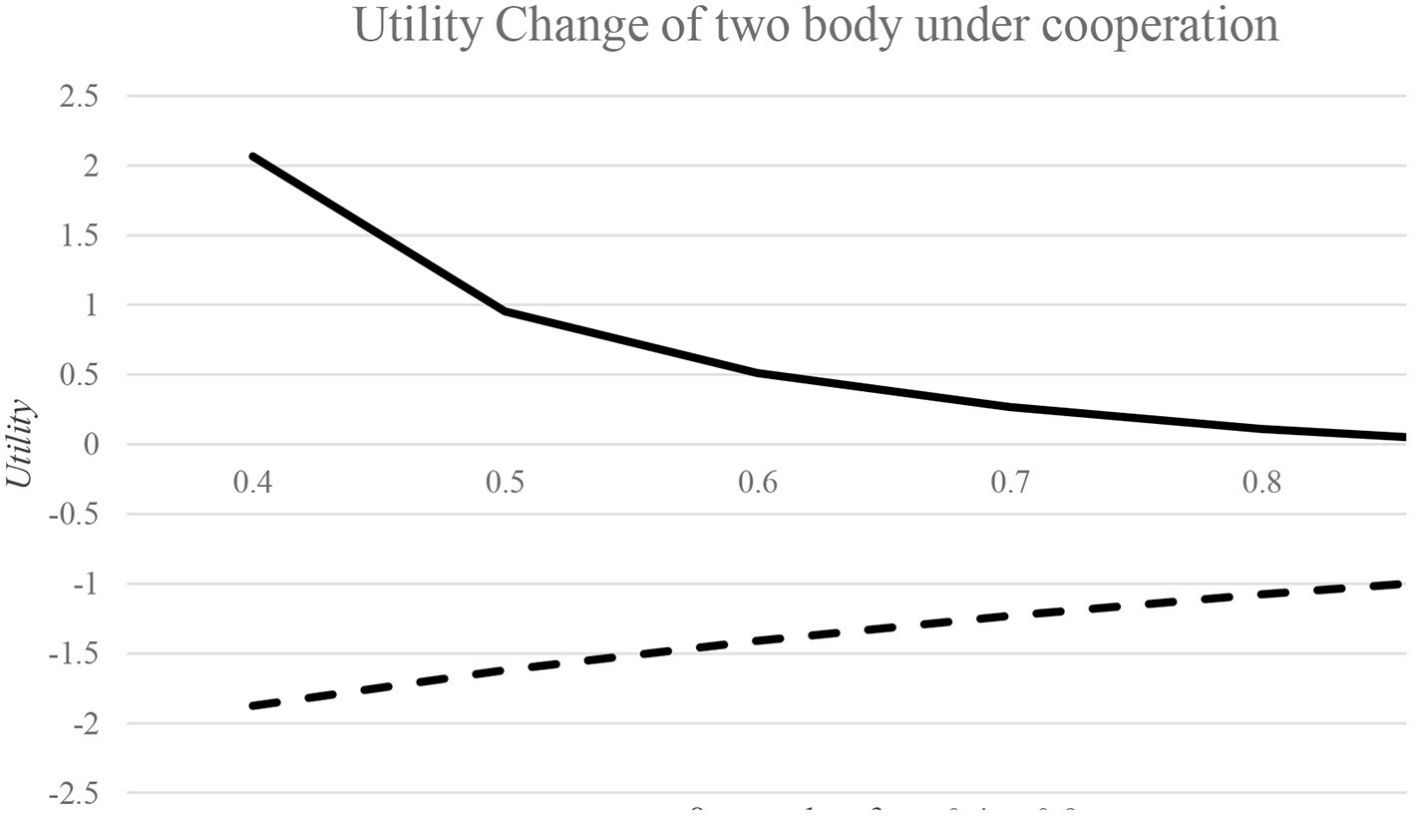
Utility change of two countries in cooperation situation with different β.

To analyze the utility change of country 1 and country 2, we need to give the specific value of the proportion of infected patients retained in their own country. Specifically, β values range from 0.4 to 0.9. The utility changes of the two countries are shown in Figure 2.

As can be seen from Figure 2, in the regional cooperation to cope with the epidemic, when other attributes of the two countries were the same, if the proportion of infected cases retained in country 1 gradually increases, that is, more and more infected patients stay in their own country, its utility loss in cooperation will be smaller and smaller, because the decline of the overall number of infected cases in the region makes it obtain the utility of controlling the number of infected persons. However, due to the strict prevention and control measures implemented by country 2, its economic losses have gradually increased and its utility in the cooperation has gradually decreased. However, on the whole, as the gap of the proportion of infected cases retained in their own country between the two countries is narrowing, the utility changes of the two countries are gradually approaching, which is easier to form the cooperation mechanism.

## Discussion

The results obtained have allowed us to discuss how to cope with the epidemic when the COVID-19 outbreaks in different countries in a region. First of all, compared with the non-cooperative situation, regional cooperation in response to the epidemic can reduce the number of infected people in the region and increase the overall regional benefit. In the process of containing the epidemic, countries cannot fight alone. The mutual flow of infected people will increase the total number of infected people in the region, reducing the total benefit in the region. Establishing a regional joint prevention and control mechanism, and restricting the flow of infected people between countries are conducive to maximize the overall benefit of the economy and epidemic prevention and control. For example, the cooperation mechanism between China, Japan, and South Korea and regional governance cooperation in Northeast Asia have achieved remarkable results in the process of preventing and controlling the epidemic.

Second, the benefits of regional cooperation for each country are different due to the differences in the attributes of the countries. The specific performance is that when the difference of preference for preventing and controlling infected cases between the two countries is small, then there is a win-win situation in the regional cooperation, that is, both countries get higher benefits in the cooperation than in the non-cooperation situation. However, when the difference of preference for preventing and controlling infected cases between the two countries exceeds the critical value, the economic cost generated by preventing and controlling the epidemic in low preference countries gradually increases, resulting in the decrease of net income and gradually generating a loss, while countries that have a high preference for containing the epidemic still obtain net income in cooperation. Therefore, when the difference in preferences for controlling the epidemic between countries are similar or tend to be the same, regional cooperation is easier to achieve, otherwise, cooperation is difficult to achieve. Meanwhile, when analyzing the impact of the cooperation mechanism on the utility of the two countries under the different proportions of infected patients flowing to the other country, we found that as the gap of the proportion of infected cases retained in own country between the two countries is narrowing, the utility changes of the two countries are gradually approaching, which makes it easier to form the cooperation mechanism. Therefore, a cooperation mechanism should be advocated among countries with similar population mobility policies during the epidemic.

Finally, the regional benefit created by cooperation should be reasonably distributed. In view of the situation that the benefit of one country is damaged in the cooperative management, we should carry out the distribution of cooperative income on the basis of its loss. The distribution of cooperative income should be based on two aspects: on the one hand, the same amount of benefit distribution is less attractive to countries with large economies but more attractive to countries with small economies; on the other hand, the preference for epidemic prevention and control reflects that a country is willing to sacrifice the economic utility to reduce the number of infected people. Countries with a low preference for prevention and control of the epidemic could pay more attention to economic development and have less motivation to participate in cooperative management. The distribution of cooperative income should be inclined to them and give them more incentives. Therefore, the distribution of cooperative income should be inclined to the countries with small economic scale and low preference for preventing and controlling infected cases, so as to promote the achievement of regional cooperation.

## Conclusion

COVID-19 has been continuing on the global scale for a long time. Isolation and stopping public activities had a negative impact on economic development. Governments of each country are facing the difficult choice of epidemic prevention and control or economic restart. Based on the two-agent game analysis, this study finds that regional cooperation to face the epidemic can maximize the regional benefits brought by containing the epidemic and improving economic performance. But at the same time, due to the differences in economic scale and preferences for epidemic prevention and control, not all of them can directly benefit from the cooperation, and the cooperative incentive of each country needs to be strengthened through the allocation of cooperative income.

## Limitations

Although the above comprehensive analysis can provide robust support for the conclusion of this study, there are still some limitations that need to be further improved. First, the premise assumption of game theory is that the players in the game make rational decisions to maximize their personal interests. However, we know that the behavior between countries will be interfered with by political system, cultural identity, and other factors, thereby affecting the applicability of the game theory model, and we only considered how to form a cooperation mechanism under the condition of two body, and the situation of more countries was not taken into account. Second, this study describes the evolution of behavior from two countries in epidemic from the perspective of building a theoretical model. Due to the lack of data, no empirical research has been carried out. If data are available, further empirical research can be carried out on this basis. Third, the model did not take into account the influence of COVID-19 in terms of the epidemiological aspect. As we know, the COVlD-19 has a strong transmission and a long incubation period. Vaccine research, wearing masks, and social isolation measures for COVID-19 have effectively prevented the increase of infection cases. The experience learned from the prevention of COVID-19 will inevitably promote epidemiological research and help us respond to future public health events. However, due to the difficulty of setting relevant indicators, the model did not consider it.

## Data Availability Statement

The original contributions presented in the study are included in the article/supplementary material, further inquiries can be directed to the corresponding author.

## Author Contributions

YW and HY conceived this research. HY, YW, and LT were responsible for the methodology. SiZ conducted software analyses. ShZ and YW conducted necessary validations. YW conducted a formal analysis and managed the investigation. ShZ and SiZ gathered resources, curated all data, wrote/prepared the original draft, and were responsible for project administration. YW and ZL reviewed and edited the manuscript, were responsible for visualization, supervised the project, and acquired funding. All the authors contributed to the article and approved the submitted version.

## Funding

This study was supported by the Humanities and Social Sciences Fund of the Ministry of Education (Grant Numbers: 19YJC790167).

## Conflict of Interest

LT was employed by company Wuhan WuXi AppTec New Drug Development Co., Wuhan, China. The remaining authors declare that the research was conducted in the absence of any commercial or financial relationships that could be construed as a potential conflict of interest.

## Publisher's Note

All claims expressed in this article are solely those of the authors and do not necessarily represent those of their affiliated organizations, or those of the publisher, the editors and the reviewers. Any product that may be evaluated in this article, or claim that may be made by its manufacturer, is not guaranteed or endorsed by the publisher.
